# Novel and Neuroprotective Tetranortriterpenoids from Chinese Mangrove *Xylocarpus granatum* Koenig

**DOI:** 10.1038/srep33908

**Published:** 2016-09-23

**Authors:** Zhen-Fang Zhou, Tibor Kurtán, Attila Mándi, Yu-Cheng Gu, Li-Gong Yao, Guo-Rong Xin, Xu-Wen Li, Yue-Wei Guo

**Affiliations:** 1 State Key Laboratory of Drug Research, Shanghai Institute of Materia Medica, Chinese Academy of Sciences, Shanghai, 201203, China; 2 Department of Organic Chemistry, University of Debrecen, POB 20, 4010 Debrecen, Hungary; 3 Syngenta Jealott’s Hill International Research Centre, Berkshire RG42 6EY, United Kingdom; 4 Institute of Biological Science, Sun Yat-Sen University, Xin Gang West Road 135, Guangzhou 510275, China

## Abstract

Eight new tetranortriterpenoids (1–8) were isolated from the twigs and leaves of the Chinese mangrove plant *Xylocarpus granatum*, together with four related known ones (9–12). The structures of new compounds were elucidated by detailed spectroscopic analysis. The absolute configuration of 9-*epi*xylogranatin A (1) was determined by time-dependent density functional theory-electronic circular dichroism (TDDFT-ECD) calculations of the solution conformers. Xylogranatumin A (2) represents the first example of the 9, 10-*seco* limonoid with an unprecedented oxygen-bridged B ring (2,7-dioxabicyclo[2.2.1]-heptane). All the isolates were evaluated for the *in vitro* neuroprotective activity, both compounds 11 and 12 displayed moderate effects against H_2_O_2_-induced neurotoxicity in PC12 cells at the concentration of 10 *μ*M, with an increase in cell viability of 12.0% and 11.6%, respectively.

The mangrove plants of the genus *Xylocarpus* (family Meliaceae) are widely distributed in the coastal areas of Southeast Asia, Australia, East Africa, and Indian Ocean. *X. granatum*, one of the three *Xylocarpus* species, has been used as a folk medicine in Southeast Asia and India for the treatment of diarrhea, cholera and fever diseases^1^,^2^. *X. granatum* was reported as a rich source of limonoids, featuring by their highly diverse and complex polycyclic skeletons^3^,^4^,^5^,^6^. These structurally interesting and challenging molecules have attracted great attention for their total synthesis, bioactivity evaluation, and biosynthetic studies^7^,^8^.

In the course of our ongoing search for bioactive substances from Chinese mangrove plants^9^,^10^,^11^,^12^,^13^, the twigs and leaves of *X. granatum* were recently collected from Hainan Province, China. A preliminary chemical investigation of them has led to the isolation and characterization of the rearranged pyridine-containing limonoids, phragmalin orthoesters, and apotirucallane protolimonoids^14^,^15^,^16^. To further explore the bioactive compounds in *X. granatum*, we continued the phytochemical analysis on the minor components in its twigs and leaves, resulting in the isolation and structure elucidation of eight new tetranortritepenoids, named 9-*epi*xylogranatin A (**1**), xylogranatumin A (**2**), 6-*O*-acetyl xylocarpin D (**3**), 14-hydroxy-14,15-dihydrogranatumin C (**4**), 30-*O*-tigloylhainangranatumin J (**5**), 9-*O*-methyl xylogranatin R (**6**), 30-*O*-acetylhainangranatumin E (**7**), and 1,2-dihydro-3*α*-hydroxy-turranolide (**8**), along with four related known compounds (**9**–**12**) (Fig. 1). Among the newly discovered natural products, xylogranatumin A (**2**) comprises an unprecedented B ring bearing an oxygen bridge between C-1 and C-8. Herein, we report the isolation, structure elucidation, and bioassay results of these compounds, as well as the plausible biosynthetic pathway of compound **2**.

## Results and Discussion

### Isolation and structure elucidation

**Figure 1:**
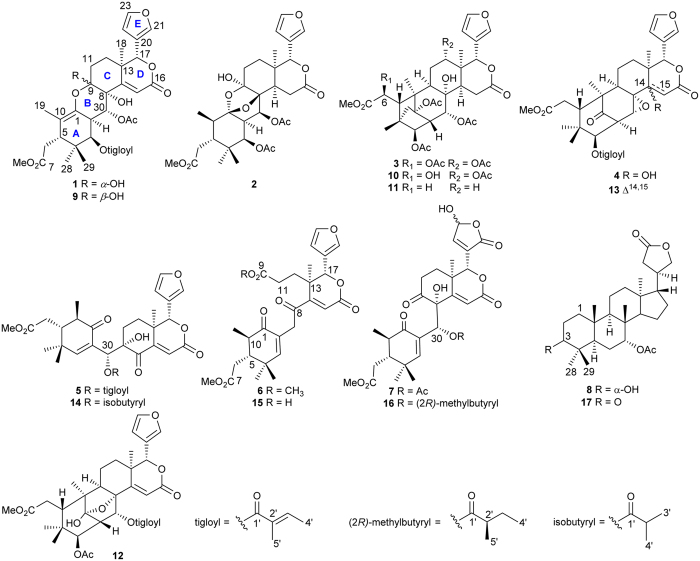
Structures of compounds 1–17.

The air-dried powdered twigs and leaves (2.0 kg) of *X. granatum* were percolated thoroughly with MeOH at room temperature. The concentrated MeOH extract was partitioned between EtOAc and H_2_O. The EtOAc-soluble portion was repeatedly chromatographed to afford twelve compounds. Four known of them were readily identified by comparison of their spectroscopic data with those reported in the literature to be xylogranatin A (**9**)^4^, xylocarpin D (**10**)^17^, xylocarpin B (**11**)^17^, and xylocarpin G (**12**) (Fig. 1).^17^ On the basis of careful analysis of NMR data, and by comparison with the known compounds, the eight new natural products were determined to be tetranortriterpenoids. Among them, **1**–**6** all showed characteristic signals of a *α*-furyl ring at C-17 position, whereas compound **7** possessed a *γ*-hydroxybutenolide group instead. At the same position, while differed from **1**–**7**, compound **8** exhibited a *γ*-butyrol lactone moiety. Accordingly, the detailed structure elucidation of these new molecules is described as follows.

**Figure 2:**
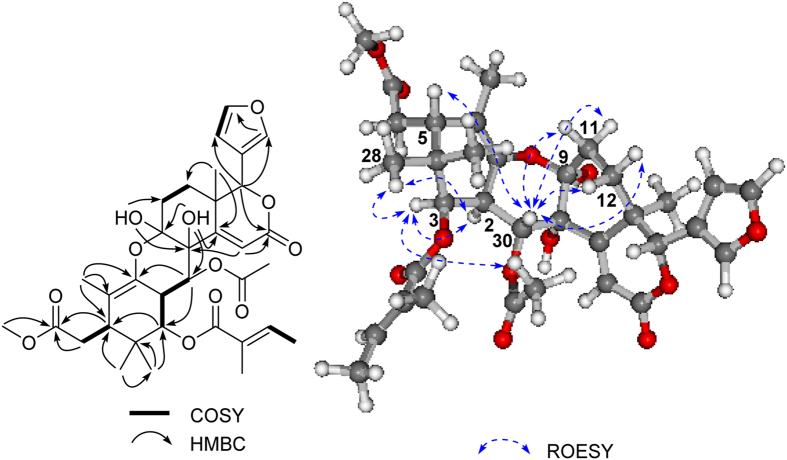
Key ^1^H-^1^H COSY, HMBC, and ROESY correlations of 1.

9-*epi*xylogranatin A (**1**) was isolated as a colorless gum. The molecular formula of **1** was determined to be C_34_H_42_O_12_ by HRESIMS (*m*/*z* 665.2554 [M + Na]^+^, calcd. 665.2574), corresponding to the molecular formula C_34_H_42_O_12_, which is the same as that of the co-occurring tetranortriterpenoid, xylogranatin A (**9**), previously isolated from the seeds of the same species.^4^ The NMR data of **1** showed diagnostic signals of a furan ring [*δ*_C_ 109.8, 119.7, 141.2, and 142.9; *δ*_H_ 6.34 (1H, d, *J* = 0.9 Hz), 7.41 (1H, s), and 7.41 (1H, t, *J* = 0.9 Hz)], an *α*, *β*-unsaturated lactone group (*δ*_C_ 117.7, 163.7, and 165.5; *δ*_H_ 6.12 s), and a tetrasubstituted double bond (*δ*_C_ 119.7, C-10 and 136.6, C-1). Detailed analysis of 1D (Tables 1 and 2) and 2D NMR (Fig. 2) spectra revealed that the planar structure of **1** was identical to that of **9**.^4^ In fact, the ^13^C NMR data from C-3 to C-7 and C-16 to C-23 of **1** are almost the same as those of **9**, and the main differences at C-1, C-2, C-8, C-9, C-10, C-12, C-15 and C-30, indicated the different stereochemistry between **1** and **9** in certain chiral centers. The relative configurations of six stereocenters at C-2, C-3, C-5, C-13, C-17, and C-30 were elucidated to be the same as those of **9** by the comparison of their ROESY spectral results, as well as by analysis of the coupling constants and splitting patterns of H-2 (*δ*_H_ 3.30, dd, *J* = 11.1, 3.8 Hz), H-3 (*δ*_H_ 5.02, d, *J* = 3.8 Hz), and H-30 (*δ*_H_ 5.10, d, *J* = 11.1 Hz). NOE correlations observed of H-2/H-3, H-2/CH_3_-28, H-3/CH_3_-28, and H-3/Ac-30 (Fig. 2) suggested that both H-2 and H-3 were *α*-oriented. On the contrary, the *β*-orientation of H-5 and H-30 was determined by the significant NOE cross-peak of H-5/H-30. The coupling constants of H-2/H-3 (*J* = 3.8 Hz) and H-2/H-30 (*J* = 11.1 Hz) indicated that both H-2 and H-30 were *axial* orientation, whereas H-3 was *equatorial* orientation, which further supported the relative configurations of H-2, H-3, and H-30. The diagnostic NOE correlations of H-30/H_2_-11 and H-30/H_2_-12 revealed that rings B and C were *cis*-fused, and both 8-OH and 9-OH were *α*-oriented. Thus, compound **1** was established as 9-epimer of xylogranatin A (**9**).

**Figure 3:**
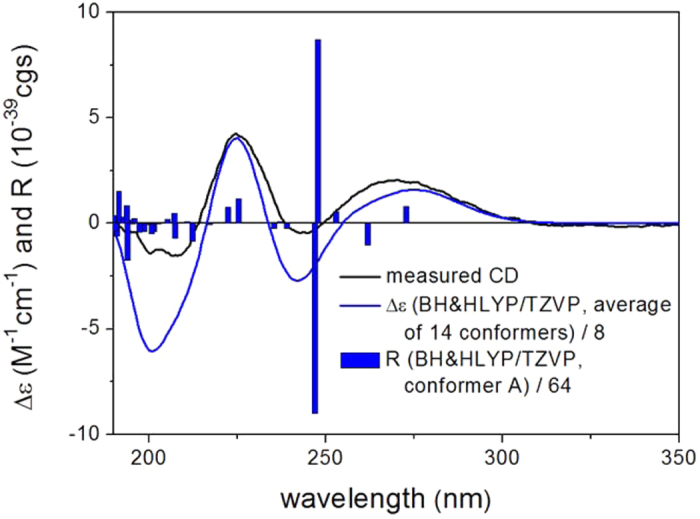
Experimental solution ECD spectrum of 9-*epi*xylogranatin A (1) and BH&HLYP/TZVP PCM/MeCN calculated ECD spectrum of (2*R*, 3*R*, 5*S*, 8*R*, 9*S*, 13*R*, 17*R*, 30*R*)-1 calculated for the low-energy solution conformers. Bars represent the calculated rotational strengths of the lowest-energy conformer.

With the aim to determine the absolute configuration of **1**, time-dependent density functional theory electronic circular dichroism calculations (TDDFT-ECD) of its solution conformers were carried out. The ECD spectrum of **1** was recorded in acetonitrile (MeCN), which showed positive, negative and positive Cotton effects (CEs) at 270, 243 and 225 nm, respectively. The initial Merck Molecular Force Field (MMFF) conformational analysis of the arbitrarily chosen (2*R*, 3*R*, 5*S*, 8*S*, 9*R*, 13*R*, 17*R*, 30*R*) absolute configuration of **1** resulted in 151 conformers, which were re-optimized at B3LYP/6-31G(d) level of theory in *vacuo*, as well as at B3LYP/TZVP level with Polarizable Continuum Model (PCM) solvent model for MeCN. The gas-phase re-optimization afforded 9 conformers with Boltzmann population above 2%, while the PCM one yielded 14 geometries above 2% (see S46 in supporting information). These structures were used as input for ECD calculations. The C-3 tigloyl group is more suitable for an axial orientation in the low-energy conformers (conformers A-H), which is in accordance with the coupling constant between H-2 and H-3 (^3^*J*_2-Hax,3-Heq_ = 3.8 Hz). Ring B possesses a twist-boat conformation with *equatorial* 30-OAc group in most of the conformers, which is in agree with the coupling constants between H-3 and H-30 (^3^*J*_3-Hax,30-Hax_ = 11.1 Hz). As to prevent the side effects of the solvent, the ECD spectra were also calculated for the (2*R*, 3*R*, 5*S*, 8*S*, 9*R*, 13*R*, 17*R*, 30*R*) enantiomer with various functions (B3LYP, BH&HLYP, PBE0) and TZVP basis set for both the in *vacuo* and the solvent model re-optimized structures. The computed ECD curves of the individual conformers were quite similar to each other, and as well as to the experimental ECD spectrum, since their rings D and E share the same orientation. Moreover, all the Boltzmann-weighted ECD spectra reproduced well the experimental ECD curve with BH&HLYP/TZVP PCM/MeCN giving the most agreement (Fig. 3). Thus, the absolute configuration of **1** was unambiguously determined as (2*R*, 3*R*, 5*S*, 8*S*, 9*R*, 13*R*, 17*R*, 30*R*). To our best knowledge, this is the first report on the configurational assignment of limonoids with 9, 10-*seco* skeleton by TDDFT ECD calculations.

The related xylogranatin A (**9**) was only reported for its relative configuration by the X-ray diffraction analysis^4^, while as it shared the same planar structure as that of 9-*epi*xylogranatin A (**1**), the absolute configuration of **9** could then be determined by comparing their ECD spectra, which rely upon the unsaturated *δ*-lactone and furan chromophores of ring D governed by the C-13 and C-17 chirality centers. The similar ECD pattern of **9** [225 nm (Δε + 23.66), 269 nm (Δε +1.57) in MeCN] and **1** indicated the same (13*R*, 17*R*) absolute configuration for both compounds. Thus the absolute configuration of xylogranatin A (**9**) can be deduced as (2*R*, 3*R*, 5*S*, 8*S*, 9*S*, 13*R*, 17*R*, 30*R*), which could further allow the absolute configurational assignment of the related xylogranatins B-D^4^.

The molecular formula of xylogranatumin A (**2**), C_31_H_40_O_12_, was deduced by HRESIMS with ion peak at m/z 627.2431 [M + Na]^+^ (calcd 624.2417), suggesting the presence of twelve degrees of unsaturation. In the ^1^H NMR spectrum, resonances arising from seven methyl groups were observed, including two geminal (*δ* 0.92 and 0.94, each 3H, s), one terminal (*δ* 1.08, 3 H, s), one secondary (*δ* 1.02, 3H, d, *J* = 6.6 Hz), one methoxyl (*δ* 3.68, 3H, s) and two acetyl groups (*δ* 2.11 and 2.15, each 3H, s). Diagnostic proton signals represent a *β*-furan ring (*δ* 6.38, 7.41, and 7.43) was also obtained. The ^13^C NMR spectrum of **2** disclosed seven methyl groups, four sp^3^ methylenes, three sp^2^ methines, seven sp^3^ methines, and ten quaternary carbons (four carbonyl groups). With regard to the 12 degree of unsaturation, and bearing in mind the presence of a furan ring and four carbonyl groups, xylogranatumin A (**2**) should possess five other rings.

**Figure 4:**
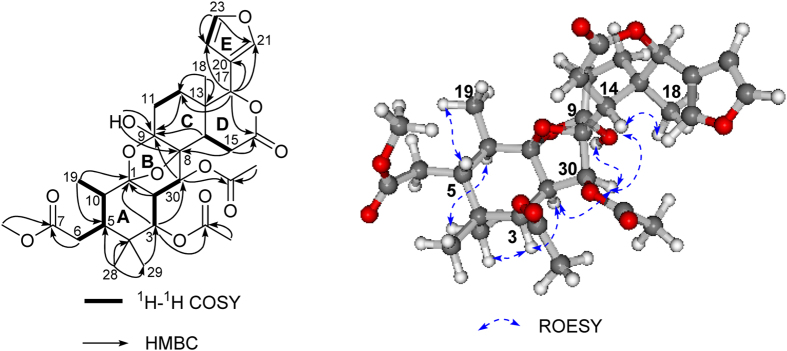
Key ^1^H-^1^H COSY, HMBC, and ROESY correlations of 2.

**Figure 5:**
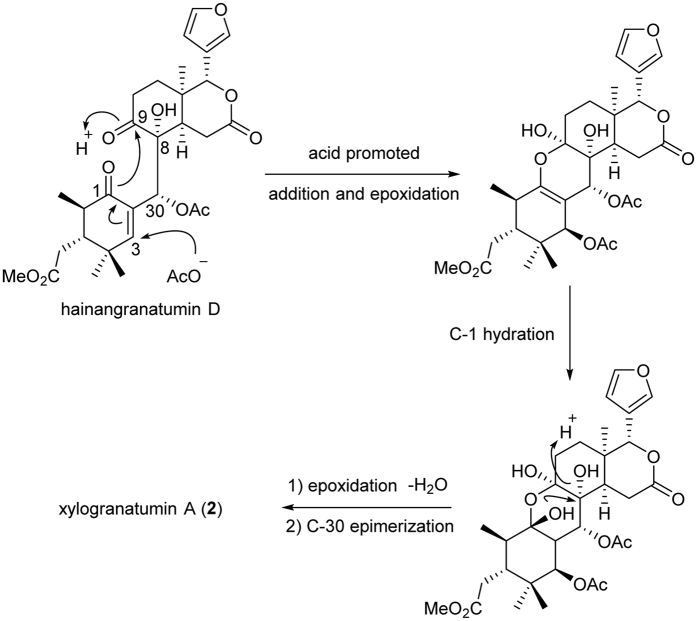
Plausible biosynthetic pathway of xylogranatumin A from hainangranatumin D.

The aforementioned data implied that compound **2** bore a similar skeleton as that of compound **1**. The comparison of the 1D and 2D NMR data of **2** with those of the co-occurring limonoid **1** indicated that they shared the similar rings C, D, and E (Fig. 4). Correspondingly, the construction of rings A and B was important for the structure determination of **2**. Two proton-bearing partial structures of C-19 → C-10 → C-5 → C-6 and C-3 → C-2 → C-30 were readily recognized from the ^1^H-^1^H COSY spectrum (Fig. 5). These two structural segments were connected to the quaternary carbons, C-1 and C-4, respectively, by the observation of HMBC correlations from Me-19 (*δ* 1.02, 3H, d, *J* = 6.6 Hz) to C-1/C-5, H-3 (*δ*_H_ 5.52, 1H, d, *J* = 3.5) to C-1/C-30, H-2 (*δ*_H_ 2.08, 1H, t, *J* = 3.5) to C-1, Me-28 to C-4/C-5 and Me-29 to C-3/C-4. In addition, the HMBC correlations of OH-9 to C-9 and C-8, as well as the typical hemiketal chemical shift of C-9 (99.8), revealed that the hydroxyl group (*δ*_H_ 3.30, OH-9) linked to C-9, which was further connected to C-8. The methoxyl group was located at C-7 on the basis of the HMBC cross-peak from MeO- to C-7, and the assignment of the two acetoxyl groups at C-3 and C-30 were clearly indicated by HMBC correlations from H-3 (*δ* 5.52, 1 H, d, *J* = 3.5 Hz) to *δ*_C_ 169.8 and H-30 (*δ* 5.30, 1 H, d, *J* = 3.5 Hz) to *δ*_C_ 170.2 (Fig. 4).

In terms of ring B, the presence of one acetal carbon at *δ*_C_ 109.2 (C-1, sp^3^), one oxygenated quaternary carbon signal at *δ*_C_ 89.6 (C-8, sp^3^), a typical hemiacetal carbon at *δ*_C_ 99.8 (C-9), and a tertiary oxygenated carbon at *δ*_C_ 71.3 (C-30) suggested that ring B was constructed *via* the C-8 → C-30 → C-2 → C-1 → O → C-9 → C-8 bond to form a tetrahydropyran ring with an oxygen bridge between C-8 and C-1. The linkage of C-30 to C-8 was also confirmed by the distinct HMBC correlation from H-30 to C-9. A planar structure of **2** was thus proposed as depicted in Fig. 1, which was consistent with its molecular composition and degrees of unsaturation.

The relative configuration of compound **2** was elucidated by the analysis of ROESY spectrum and proton coupling constants, as well as by analogy with that of **1**. The same relative stereochemistry of C-2, C-3, C-5, C-13 and C-17 in **2** was deduced from the similar carbon chemical shifts, proton coupling constants, and ROESY correlations with those of **1** and the known compounds **10**–**16** (for C-13 and C-17), as well as by a biogenetic consideration of such limonoids in Nature^2^,^17^. In addition, the NOE correlations between H-5 and CH_3_-19, H-10 and CH_3_-28, CH_3_-28 and H-3, H-3 and H-2, H-14 and CH_3_-18, H-14 and H-30, H-30 and OH-9 (Fig. 4) further confirmed the relative configuration of 2R*, 3R*, 5S*, 9S*, 10R*, 13R*, 14R*, 17R*, 30S*. Although the relative configurations of C-1 and C-8 cannot be determined by distinct ROE correlations, the correlation between H-30 and OH-9 implied the *β* -orientation of the oxygen bridge due to the smaller transannular strain.

Biogenetically, this interesting molecule might be derived from hainangranatumin D (Fig. 5), a limonoid previously isolated from *X. granatum* with absolute configuration established^18^, by a first plausible weak acid promoted nucleophilic addition of acetoxyl group at C-3 position, which allowed the double bond migration and the epoxidation from C-1 to C-9. The resulting intermediate then underwent a C-1 hydration, followed by a second acid promoted epoxidation from C-1 to C-8 with the elimination of H_2_O. Finally, a C-30 epimerization, which possibly occurred during the previous epoxidation to lower the energy of the molecule, allowed the production of compound **2**. Since the relative stereochemistry has been established, the common biosynthetic origin of **2**, compound **1**, the known compounds **9**–**16** and hainangranatumin D^2^,^4^,^17^,^18^, suggested the corresponding chiral centers, especially C-13 and C-17 adjacent to the furan core should be the same. Therefore, the absolute configuration of compound **2** was deduced as (1*S*, 2*R*, 3*R*, 5*S*, 8*S*, 9*S*, 13*R*, 17*R*, 30*S*). Based on the above information, xylogranatumin A (**2**) was determined as a novel limonoid characterized by a 9, 10-*seco* skeleton bearing an oxygen-bridge between C-1 and C-8 (Fig. 1). and the discovery of xylogranatumin A provided a new example to the extremely diverse and complex family of limonoids.

6-*O*-acetyl xylocarpin D (**3**) gave a HRESIMS pseudomolecular ion peak at m/z 769.2634 [M + Na]^+^, a plus of 42 mass units on that of the co-occurring xylocarpin D (**10**), which was previously isolated from the fruits of *X. granatum* with absolute configuration determined^17^, indicating the presence of an additional acetyl group in **3**, which was further confirmed by the careful comparison of their ^1^H and ^13^C NMR data (Tables 1 and 2), with an observation of additional peaks of *δ*_H_ at 2.19 and *δ*_C_ at 169.6 and 21.0 on **3**. The location of the acetyl group at C-6 was established by the expected downfield shifted ^1^H NMR resonance of H-6 (from *δ*_H_ 4.29 to 5.30). Therefore, compound **3** was determined as the 6-acetyl derivative of xylocarpin D (**10**).

The HRMS data for 14-hydroxy-14,15-dihydrogranatumin C (**4**) displayed a pseudomolecular ion peak at 584.2643 [M]^+^ (calcd 584.2621), consistent with the molecular formula C_32_H_40_O_10_. Detailed analysis of the ^1^H and ^13^C NMR data of **4** were reminiscent of those of **13**, which was previously isolated from the seeds of a Krishna mangrove, *X. granatum*^19^. Their main differences were an oxygenated quaternary carbon at C-14 (*δ*_C_ 62.7) and a methylene carbon at C-15 (*δ*_C_ 40.8; *δ*_H_ 3.53, 1H, d, *J* = 16.0 Hz; 2.75, 1H, d, *J* = 16.0 Hz) in **4** in replace of the Δ^14^,^15^ double bond (*δ*_C_ 160.9 and 118.8) in **13**. These data indicated that the Δ^14^,^15^ double bond was hydrated in **4**, which is in full agreement with a plus of eighteen mass units of **4** on that of **13**. The HMBC correlations from H-15 to C-14 and C-16, and CH_3_-18 to C-14 gave further support to the structural assignment of **4**.

The relative configuration of **4**, except C-14 position, was determined to be the same as that of **13** due to the similar ^13^C NMR shifts and coupling constants in ^1^H NMR and further confirmed by ROESY experiments. Unfortunately, the absence of the proton signal of OH-14 in **4**, which were usually presented in the NMR spectra of the limonoids when measuring in CDCl_3_, such as Granaxylocarpin C^20^, bearing the similar substructure as compound **4**, prevented us from assigning the configuration of C-14 position via the available NMR data.

30-*O*-tigloylhainangranatumin J (**5**) was isolated as an optically active white amorphous powder. The molecular formula, C_32_H_38_O_10_, was established by HRESIMS from the ion peak at 605.2346 [M+Na]^+^. Its ^1^H and ^13^C NMR data (Tables 1 and 2) were closely related to those of hainangranatumin J (**14**)^18^. The only difference was the replacement of the 30-*O*-isobutyryl group in **14** by a tigloyl moiety (*δ*_H_ 6.78, q, *J* = 6.8 Hz, 1.80, d, *J* = 6.8 Hz, and 1.77, s; *δ*_C_ 166.6 qC, 127.6 qC, 139.8 CH, 14.6 CH_3_, and 11.9 CH_3_) in **5**. Therefore, the structure of 30-*O*-tigloylhainangranatumin J (**5**) was determined as shown in Fig. 1.

The molecular formula of 9-*O*-methyl xylogranatin R (**6**), C_28_H_34_O_9_, was deduced by HRESIMS (*m*/*z* 537.2100, calcd for [M+Na]^+^ 537.2101). The ^1^H and ^13^C NMR spectra of **6** were almost identical to those of xylogranatin R (**15**), which was isolated as an antifeedant from the seeds of the same species with the absolute configuration established^5^, except for the presence of an additional methoxy group (*δ*_H_ 3.68; *δ*_C_ 51.8), suggesting that **6** was an *O*-methyl derivative of **15**, in agreement with an addition of 14 mass units in **6** to that of **15**. The chemical shift of C-9 (*δ* 172.8) in **6** was upfield shifted Δ*δ* 2.7 from that of **15**, indicating the carboxylic acid at C-9 was esterified to methyl ester, which was further confirmed by the HMBC correlation from 9-OCH_3_ (*δ*_H_ 3.67) to the carbonyl carbon at C-9 (*δ*_C_ 172.8). The complete assignments of the ^1^H and ^13^C NMR of **6** were achieved by a comprehensive analysis of 2D NMR spectra including HSQC, COSY, HMBC, and ROESY. Compound **6** was thus determined as the methyl ester of xylogranatin R (**15**). In view of the presence of methyl ester moiety in a great number of limonoids previously isolated from this species, such as xylogranatins A-D^4^, and hainangranatumins A-J^18^, the authors believe that compound **6** is an original natural product rather than an artifact.

The molecular formula of 30-*O*-acetylhainangranatumin E (**7**) was established as C_29_H_34_O_12_ by HRMS (*m*/*z* 597.1951, calcd for [M+Na]^+^ 597.1948). The *γ*-hydroxybutenolide group was characterized by proton signals at *δ*_H_ 7.42 (H-22) and 6.24 (H-23), and by carbon signals at *δ*_C_ 132.9 (C-20), 169.1/168.8 (C-21), 150.4/149.7 (C-22), and 97.4/96.7 (C-23) in its ^1^H and ^13^C NMR spectra (Tables 1 and 2). The detailed NMR data analysis reminded us those of hainangranatumin E (**16**), previously isolated from the seeds of Hainan mangrove *X. granatum*^18^. The only differences were the presence of the acetyl group (*δ*_H_ 2.09 s; *δ*_C_ 169.9/169.8 qC, 21.0 CH_3_) at C-30 in **7** instead of the methylbutyryl group in **16**. In addition, two sets of carbon resonances at *δ* 208.2/208.0 (C-9), 38.6/38.5 (C-13), 164.5/164.3 (C-16), 78.2/77.9 (C-17), 18.4/18.3 (C-18), 169.1/168.8 (C-21), 150.4/149.7 (C-22), 97.4/96.7 (C-23) in ^13^C NMR spectrum suggested compound **7** was a mixture of unseparated C-23 epimers as depicted in Fig. 1.

1,2-dihydro-3α-hydroxy-turranolide (**8**) was isolated as a colorless gum. Its molecular formula, C_28_H_42_O_5_, was deduced by HREIMS at *m*/*z* 481.2892, a plus of two mass units to that of turranolide (**17**), previously isolated from the root bark of *Turraea robusta* collected from Awasi, Kisumu District, Kenya^21^. The NMR spectra of **8** were similar to those of **17**, except for the presence of oxymethine signal at C-3 (*δ*_H_ 3.42, t, *J* = 2.8 Hz) in ^1^H NMR. A reduction of the ketone at C-3 in **17** to the hydroxyl group in **8** was then easily recognized, which further confirmed by the upfield ^13^C NMR signal at 75.9 in **8** in replace of that at *δ* 216.2 in **17**. In addition, the *α*-orientation of the hydroxyl at C-3 of **8** was confirmed by the ROESY correlations of H-3/CH_3_-29 and H-5/CH_3_-28. The structure of 1,2-dihydro-3α-hydroxy-turranolide (**8**) was thus determined as the C-3 reductive derivative of turranolide (**17**).

Similar as that of compound **2**, since the relative stereochemistry of the new compounds **3**, **5**, **6**, and **8** has been established, the common biosynthetic origin of such furan limonoids^2^,^4^,^17^,^18^ suggested the corresponding chiral centers, such as C-13 and C-17 should be the same *R* configuration. Thus the absolute configuration of the above mentioned new compounds could be arbitrary determined as showed in Fig. 1.

In summary, eight new tetranortriterpenoids (**1**–**8**) together with four related known compounds (**9**–**1**2) were isolated from the twigs and leaves of Chinese mangrove plant *X. granatum*. The structures of new compounds were elucidated by extensive spectroscopic analysis. The absolute configuration of 9-*epi*xylogranatin A (**1**) was determined by TDDFT ECD calculations, which incidentally allowed the elucidation of the absolute configurations of xylogranatin A (**9**) by comparison with their ECDs, solving a puzzle of the previously reported natural products. The discovery of the 9, 10-*seco* limonoid **2** with a characteristic 2,7-dioxabicyclo [2.2.1]-heptane ring system added a novel skeleton to the family of tetranortriterpenoids, revealing the high diversity and complexity of such beautiful molecules.

### Neuroprotective activity evaluation

In the light of a wide range of biological activities and pharmacological properties of limonoids^2^,^8^, we performed *in vitro* investigation of neuroprotective activity of compounds 1–12 on PC12 cells, since the isolated protolimoloids by us from *Toona ciliata* var. *pubescens* displayed significant cell protecting activity^11^. Both compounds **11** and **12** showed moderate neuroprotective effects against H_2_O_2_-induced neurotoxicity in PC12 cells at the concentration of 10 *μ*M, with an increase in cell viability of 12.0% and 11.6%, respectively. N-Acetyl-L-cysteine (NAC) was used as the positive control with the increase in cell viability of 22.0% at 10 *μ*M. In comparison with the tested structures, it is possible that the variation of rings A and B of these limonoids play an important role for the neuroprotective activity.

## Methods

### General experimental procedures

Optical rotations were measured on a Perkin-Elmer polarimeter 341. CD spectra were obtained on a JASCO 810 spectrometer. IR spectra were recorded on a Nicolet-Magna FT-IR750 spectrometer. The NMR spectra were measured on Bruker DRX 400 and Varian Inova 600 spectrometers. Chemical shifts (*δ*) are reported with the residual CDCl_3_ (*δ*_H_ = 7.26 ppm) as the internal standards for the ^1^H NMR spectroscopy, and CDCl_3_ (*δ*_C_ = 77.0 ppm) for the ^13^C NMR spectroscopy. Chemical shifts were expressed in *δ* (ppm) and coupling constants (*J*) in Hz. ^1^H and ^13^C NMR assignments were supported by ^1^H–^1^H COSY, HSQC, HMBC and ROESY experiments. ESIMS and HRESIMS spectra were recorded on a Q-TOF Micro LC-MS-MS mass spectrometer. Reversed-phase HPLC analysis was performed on an Agilent 1100 series liquid chromatography using a VWD G1314A detector at 210 nm and a semi-preparative ZORBAX ODS column (250 mm × 9.4 mm i.d., 5 mm particle size. Commercial silica gel (Qing Dao Hai Yang Chemical Group Co., 200–300 mesh) was used for column chromatography (CC), and precoated silica gel plates (Yan Tai Zi Fu Chemical Group Co., G60 F-254) were used for analytical TLC.

### Plant materials

The twigs and leaves of *X. granatum* (2.0 kg) were collected in December 2009 from Dongzhai Harbor, Hainan province, China, and identified by Professor Guo-Rong Xin of Institute of Biological Science, Sun Yat-Sen University. A voucher specimen (NO. 09-P-69) is available for inspection at the Herbarium of Shanghai Institute of Materia Medica, Chinese Academy of Sciences.

### Extraction and isolation

The air-dried powdered twigs of *X. granatum* were percolated with MeOH (three times, each 7 days) at room temperature. The extract was evaporated to dryness under reduced pressure to give 154 g of residue. The residue was partitioned with EtOAc to afford 29.7 g EtOAc extract. The EtOAc extract was separated by Sephadex LH-20, MCI and silica gel column to obtain five fractions (1–5). Fr.3 was subjected to silica gel liquid chromatography, eluting with CHCl_3_/MeOH (from 100:1 to 8:2, gradient), to obtain 15 fractions (3A-3O). Fraction 3C (65.3 mg) was chromatographed on silica gel with petroleum ether/acetone (8:2 to 1;1, gradient) to yield **7** (8.6 mg), and the sub-fraction 3C1. Sub-fraction 3C1 was purified by HPLC (75:25) to afford **5** (1.8 mg). Fraction 3D was separated by silica gel column with the eluent of petroleum ether/acetone (8:2 to 7:3, gradient) and subsequently purified by semi-preparative-HPLC eluting with MeOH/H_2_O (70:30) to afford **4** (2.1 mg) and **6** (2.3 mg). Fraction 3E (2.17 g) was subjected to column chromatography on silica gel eluted with a gradient of petroleum ether/acetone (9:1 to 1:1) to give nine major fractions 3E0–3E9. 3E7 was separated by silica gel column eluted with petroleum ether/acetone (8:2 to 7:3) to afford **12** (30.0 mg) and the sub-fraction 3E7a, which was purified by reversed phase HPLC (CH_3_CN/H_2_O, 67:33) to yield **8** (5.4 mg), while compounds **3** (1.0 mg) and **11** (4.8 mg) was prepared by column chromatography on silica gel eluted with petroleum ether/acetone (7:3) from fraction 3E9. Fractions 3G was first separated by column chromatography on silica gel eluted with petroleum ether/acetone (8:2 to 6:4, gradient) to give six sub-fractions (3G1-3G6). 3G3 was purified by reversed phase HPLC eluted with MeOH/H_2_O (80: 20) to yield **1** (1.5 mg). 3G4 was separated by reversed phase HPLC (CH_3_CN/H_2_O, 50:50), then subjected to column chromatography on silica gen eluted with petroleum ether/acetone (8:2) to give **2** (2.6 mg). Fractions 3G5 and 3G6 were separated by silica gel column chromatography with petroleum ether/acetone (70:30) and (65:25) to yield **9** (4.7 mg) and **10** (21.6 mg), respectively.

### Chemical structure data

All investigated compounds were ≥95% pure (HPLC, wavelength = 210 nm).

The NMR spectra of the compounds are provided in the Supporting Information.

9-*epi*xylogranatin A (**1**). Colorless gum, 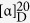 +48 (*c* 0.12, CH_3_CN); ECD (CH_3_CN) *λ*_max_ (*Δε*): 270 (2.23), 243 (−0.26), 225 (4.45), 206 (−1.53), 200sh (−1.43). IR (KBr) *ν*_max_ 3432, 2924, 1725, 1381, 1261 cm^−1^; for ^1^H NMR and ^13^C NMR spectroscopic data, see Tables 1 and 2; HREIMS *m*/*z* [M+Na]^+^ 665.2554 (calcd. for C_34_H_42_O_12_Na, 665.2574).

Xylogranatumin A (**2**). Colorless gum, 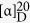 −15.0 (*c* 0.08, CH_3_CN); ECD (CH_3_CN) *λ*_max_ (*Δε*): 254 (0.12), 235 (−0.51), 212 (1.12). IR (KBr) *ν*_max_ 3436, 2925, 1736, 1374, 1262, 1162, 1036 cm^−1^; for ^1^H NMR and ^13^C NMR spectroscopic data, see Tables 1 and 2; HREIMS *m*/*z* [M+Na]^+^ 627.2431 (calcd. for C_34_H_42_O_12_Na, 627.2417).

6-*O*-acetyl xylocarpin D (**3**). White, amorphous powder, [α]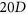 +4.3 (*c* 0.07, CH_3_CN); UV (MeOH) *λ*_max_ 213 nm. IR (KBr) *ν*_max_ 3426, 2924, 2853, 1741, 1373, 1234, 1041 cm^−1^; for ^1^H NMR and ^13^C NMR spectroscopic data, see Tables 1 and 2; HREIMS *m*/*z* [M+Na]^+^ 769.2634 (calcd. for C_34_H_42_O_12_Na, 769.2684).

14-Hydroxy-14,15-dihydrogranatumin C (**4**). White, amorphous powder, 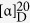 +122.5 (*c* 0.04, MeOH); UV (MeOH) *λ*_max_ 212 nm. IR (KBr) *ν*_max_ 3438, 2962, 1733, 1262, 1098, 1024, 802 cm^−1^; for ^1^H NMR and ^13^C NMR spectroscopic data, see Tables 1 and 2; HREIMS *m*/*z* [M+Na]^+^ 584.2643 (calcd. for C_32_H_40_O_10_Na, 584.2621).

30-*O*-tigloylhainangranatumin J (**5**). White, amorphous powder, 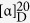 +22.2 (*c* 0.09, MeOH); UV (MeOH) *λ*_max_ 210 nm. IR (KBr) *ν*_max_ 3439, 2962, 1735, 1671, 1383, 1260, 1166, 1026 cm^−1^; for ^1^H NMR and ^13^C NMR spectroscopic data, see Tables 1 and 2; HREIMS *m*/*z* [M+Na]^+^ 605.2346 (calcd. for C_32_H_40_O_10_Na, 605.2343).

9-*O*-methyl xylogranatin R (**6**). White, amorphous powder, 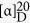 +49.1 (c 0.05, MeOH); UV (MeOH) *λ*_max_ 211 nm. IR (KBr) *ν*_max_ 3432, 2919, 1735, 1630, 1091, 1046 cm^−1^; for 1H NMR and 13C NMR spectroscopic data, see Tables 1 and 2; HREIMS m/z [M+Na]^+^ 537.2100 (calcd. for C_28_H_34_O_9_Na, 537.2101).

30-*O*-acetylhainangranatumin E (**7**). Colorless gum, 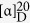 +38.9 (*c* 0.19, MeOH); UV (MeOH) *λ*_max_ 206 nm. IR (KBr) *ν*_max_ 3437, 2965, 1735, 1671, 1373, 1262, 1230, 1018 cm^−1^; for ^1^H NMR and ^13^C NMR spectroscopic data, see Tables 1 and 2; HREIMS *m*/*z* [M+Na]^+^ 597.1951 (calcd. for C_29_H_34_O_12_Na, 597.1948).

1,2-Dihydro-3α-hydroxy-turranolide (**8**). Colorless gum, 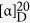 −17.6 (*c* 0.11, MeOH); IR (KBr) *ν*_max_ 3437, 2923, 1782, 1726, 1378, 1259, 1033 cm^−1^; for ^1^H NMR and ^13^C NMR spectroscopic data, see Tables 1 and 2; HREIMS *m*/*z* [M+Na]^+^ 481.2892 (calcd. for C_28_H_42_O_5_Na, 481.2930).

### Neuroprotective bioassay

The neuroprotective activities of compounds **1**–**12** against hydrogen peroxide (H_2_O_2_)-induced neurotoxicity in PC12 cells were evaluated by using the MTT method^22^, according to the protocols described in previous literature.

### Computational section

Mixed torsional/low mode conformational searches were carried out by means of the Macromodel 9.7.223 software^23^ using Merck Molecular Force Field (MMFF) with an implicit solvent model for chloroform. Reoptimizations at B3LYP/6-31G(d) level of theory in vacuo as well as B3LYP/TZVP with PCM solvent model for MeCN were carried out followed by TDDFT-ECD calculations using various functionals (B3LYP, BH&HLYP, PBE0) and TZVP basis set of the Gaussian 09 package^24^. Boltzmann distributions were estimated from the ZPVE corrected B3LYP energies in the gas-phase calculations and from the B3LYP energies in the PCM ones. ECD spectra were generated as the sum of Gaussians^25^ with 2400 and 3000 cm^−1^ half-height width (corresponding to ca. 16 and 20 nm at 260 nm, respectively), using dipole-velocity computed rotational strengths. The MOLEKEL software package^23^ was used for visualization of the results.

## Additional Information

**How to cite this article**: Zhou, Z.-F. *et al*. Novel and Neuroprotective Tetranortriterpenoids from Chinese Mangrove *Xylocarpus granatum* Koenig. *Sci. Rep.* **6**, 33908; doi: 10.1038/srep33908 (2016).

## Supplementary Material

Supplementary Information

## Figures and Tables

**Table 1:** ^1^H NMR spectroscopic data for compounds 1–8^a^.

No.	**1**	**2**	**3**	**4**	**5**	**6**	**7**	**8**
1								1.37 m
2	3.30 dd (11.1, 3.8)	2.08 t (3.5)	2.99 dd (11.6, 2.4)	3.64 dd (9.5, 2.7)				1.96 m
								1.57 m
3	5.02 d (3.8)	5.52 d (3.5)	5.28 d (11.6)	5.06 d (9.5)	6.92 s	6.48 s	6.97 s	3.42 t (2.8)
5	2.93 d (8.9)	2.24 m	3.15 s	3.38 dd (9.2, 2.3)	2.32 m	2.37 m	2.27 m	1.84 dd (12.9, 2.0)
6	2.38 dd (16.7, 3.3)	2.39 dd (16.4, 3.0)	5.30 s	2.36 m	2.32 m	2.49 d (14.3)	2.47 m	1.63 m
	2.30 dd (16.7, 8.9)	2.17 m		1.77 m	2.46 m	2.31 m	2.27 m	
8			2.24 m					
9			2.19 m	2.15 m				2.02 m
10		1.97 m			2.40 m	2.37 m	2.27 m	
11	2.19 m	2.10 m	2.05 m	2.01 m	1.79 m	2.37 m	3.10 dd (19.6, 7.2)	1.74 m
	2.03 m	1.90 m	1.86 m	1.77 m	1.67 m		2.54 m	1.47 m
12	1.73 m	1.67 m	5.09 s	1.89 m	2.29 m	2.37 m	2.88 m	1.61 m
	1.36 m	1.67 m		1.47 m	1.38 m	1.78 m	1.62 m	1.47 m
14		2.59 dd (12.7, 5.7)	2.40 d (8.6)					
15	6.12 s	2.75 dd (16.5, 12.7)	3.35 d (19.4)	3.53 d (16.0)	6.28 s	6.68 s	6.17 s	5.23 dd (3.3, 1.5)
		2.48 dd (16.5, 5.6)	2.65 dd (19.4, 8.6)	2.75 d (16.0)				
16								2.11 dd (7.4, 3.4)
17	5.19 s	5.11 s	5.75 s	5.29 s	5.52 s	5.34 s	5.33 s	1.63 m
18	1.34 s	1.08 s	0.99 s	0.90 s	1.08 s	1.19 s	1.00 s	1.01 s
19	1.67 s	1.02 d (6.6)	1.14 s	1.08 s	1.15 d (5.7)	1.07 d (6.1)	1.04 d (5.6)	0.90 s
20								2.70 ddd (13.8,10.4, 2.8)
21	7.41 s	7.41 s	7.46 s	7.61 s	7.61 s	7.52 s		4.46 t (8.2)
								3.91 t (8.2)
22	6.34 d (0.9)	6.38 s	6.37 s	6.50 d (1.4)	6.50 brs	6.44 d (0.9)	7.42 s	2.51 dd (17.1, 7.9)
								2.20 dd (17.1, 11.4)
23	7.41 t (0.9)	7.43 d (1.5)	7.45 s	7.42 t (1.4)	7.44 s	7.44 t (1.5)	6.24 s	
28	0.91 s	0.94 s	1.00 s	0.83 s	1.09 s	1.09 s	1.20 s	0.83 s
29	0.88 s	0.92 s	2.46 d (10.5)		1.14 s	1.18 s	1.11 s	0.85 s
			2.26 d (10.5)	0.83 s				
30	5.10 d (11.1)	5.30 d (3.5)	5.42 d (2.6)	3.51 d (2.7)	6.26 s	3.65 d (16.6)	6.49 s	1.09 s
						3.45 d (16.6)		
7-OMe	3.71 s	3.68 s	3.76 s	3.74 s	3.69 s	3.70 s	3.69 s	
9-OMe						3.68 s		
7-OAc								1.97 s
1-OAc			2.15 s					
3-OAc		2.15 s	2.11 s					
6-OAc			2.19 s					
12-OAc			2.10 s					
30-OAc	2.03 s	2.11 s	2.06 s				2.09 s	
3′	6.78 dd (7.2, 1.8)			7.02 m	6.78 q (6.8)			
4′	1.84 d (7.2)			1.86 d (7.0)	1.81 d (7.8)			
5′	1.84 s			1.92 s	1.77 s			
8-OH	2.95 s		3.69 brs		3.83 s		3.94 s	
9-OH	3.84 s	3.30 s						

^a^Spectra measured at 400 MHz in CDCl_3_.

**Table 2:** ^13^C NMR spectroscopic data for compounds 1–8^a^.

No.	1	2	3	4	5	6	7	8
1	136.6 qC	109.2 qC	87.8 qC	214.5 qC	198.7 qC	199.3 qC	198.6 qC	32.6 CH_2_
2	36.2 CH	55.0 CH	46.3 CH	48.6 CH	130.4 qC	129.2 qC	128.4 qC	25.0 CH_2_
3	73.7 CH	76.3 CH	75.8 CH	77.1 CH	160.9 CH	158.3 CH	161.9 CH	75.9 CH
4	37.1 qC	39.4 qC	45.2 qC	39.4 qC	36.6 qC	36.8 qC	36.8 qC	36.9 qC
5	40.4 CH	41.7 CH	42.5 CH	42.4 CH	45.2 CH	43.0 CH	45.2 CH	41.8 CH
6	32.7 CH_2_	33.6 CH_2_	71.6 CH	33.3 CH_2_	34.8 CH_2_	34.7 CH_2_	34.6 CH_2_	23.2 CH_2_
7	174.5 qC	174.0 qC	170.0 qC	174.1 qC	173.5 qC	173.6 qC	173.4 qC	75.3 CH
8	75.5 qC	89.6 qC	73.3 qC	62.7 qC	201.0 qC	197.8 qC	80.1 qC	42.2 qC
9	98.2 qC	99.8 qC	31.0 CH	54.4 CH	79.8 qC	172.8 qC	208.2/208.0 qC	43.0 CH
10	119.7 qC	36.5 CH	47.2 qC	48.2 qC	43.2 CH	45.6 CH	42.8 CH	37.4 qC
11	28.2 CH_2_	33.0 CH_2_	28.8 CH_2_	18.5 CH_2_	29.5 CH_2_	29.4 CH_2_	32.9 CH_2_	16.2 CH_2_
12	25.5 CH_2_	27.1 CH_2_	71.0 CH	33.1 CH_2_	28.6 CH_2_	29.8 CH_2_	25.6 CH_2_	33.8 CH_2_
13	38.3 qC	36.1 qC	39.3 qC	40.3 qC	41.5 qC	41.4 qC	38.6/38.5 qC	46.6 qC
14	163.7 qC	35.6 CH	46.4 CH	71.8 qC	156.6 qC	158.9 qC	162.5 qC	159.6 qC
15	117.7 CH	29.0 CH_2_	28.0 CH_2_	40.8 CH_2_	121.2 CH	124.4 CH	117.7 CH	118.0 CH
16	165.5 qC	171.2 qC	169.7 qC	169.5 qC	163.4 qC	163.4 qC	164.5/164.3 qC	34.8 CH_2_
17	81.1 CH	79.4 CH	76.8 CH	78.4 CH	81.4 CH	78.5 CH	78.2/77.9 CH	58.1 CH
18	18.8 CH_3_	24.4 CH_3_	18.8 CH_3_	20.1 CH_3_	18.6 CH_3_	20.4 CH_3_	18.4/18.3 CH_3_	19.3 CH_3_
19	12.8 CH_3_	10.8 CH_3_	22.6 CH_3_	16.4 CH_3_	12.2 CH_3_	11.7 CH_3_	11.6 CH_3_	15.4 CH_3_
20	119.7 qC	120.9 qC	120.7 qC	120.1 qC	119.2 qC	119.6 qC	132.9 qC	37.5 CH
21	141.2 CH	140.8 CH	140.4 CH	141.2 CH	141.4 CH	141.6 CH	169.1/168.8 qC	72.6 CH_2_
22	109.8 CH	109.9 CH	109.1 CH	110.4 CH	109.7 CH	109.8 CH	150.4/149.7 CH	34.1 CH_2_
23	142.9 CH	143.3 CH	143.9 CH	142.9 CH	143.3 CH	143.4 CH	97.4/96.7 CH	176.8 qC
28	24.8 CH_3_	24.4 CH_3_	15.6 CH_3_	23.0 CH_3_	20.6 CH_3_	20.1 CH_3_	27.8 CH_3_	21.9 CH_3_
29	20.1 CH_3_	20.9 CH_3_	41.0 CH_2_	20.7 CH_3_	28.0 CH_3_	28.2 CH_3_	20.6 CH_3_	28.0 CH_3_
30	68.9 CH	71.3 CH	69.7 CH	62.8 CH	66.8 CH	41.3 CH_2_	67.8 CH	27.5 CH_3_
7-OMe	52.0 CH_3_	51.9 CH_3_	53.0 CH_3_	52.4 CH_3_	52.0 CH_3_	52.0 CH_3_	52.0 CH_3_	
9-OMe						51.8 CH_3_		
7-OAc								21.3 CH_3_
								170.4 qC
1-OAc			21.0 CH_3_					
			168.4 qC					
3-OAc		21.2 CH_3_	21.1 CH_3_					
		169.8 qC	169.4 qC					
6-OAc			21.0 CH_3_					
			169.6 qC					
12-OAc			22.1 CH_3_					
			169.7 qC					
30-OAc	20.7 CH_3_	20.9 CH_3_	21.4 CH_3_				21.0 CH_3_	
	170.5 qC	170.2 qC	170.7 qC				169.9/169.8 qC	
1′	167.3 qC			167.0 qC	165.7 qC			
2′	128.7 qC			127.4 qC	127.8 qC			
3′	137.2 CH			140.4 CH	138.9 CH			
4′	14.5 CH_3_			14.8 CH_3_	14.6 CH_3_			
5′	12.1 CH_3_			12.3 CH_3_	12.0 CH_3_			

^a^Spectra measured at 100 MHz in CDCl_3._
