# Supplementary material for: Novel and Neuroprotective Tetranortriterpenoids from Chinese Mangrove Xylocarpus granatum Koenig
Source: Sci Rep. 2016 Sep 23;6:33908. doi: 10.1038/srep33908 (PMC5034266; doi:10.1038/srep33908)
Supplement: Supplementary Information [file srep33908-s1.doc]

**Supplementary data**

Novel and Neuroprotective Tetranortriterpenoids from Chinese Mangrove *Xylocarpus granatum* Koenig

Zhen-Fang Zhou,^1^ Tibor Kurtán,^2^ Attila Mándi,^2^ Yu-Cheng Gu,^3^ Li-Gong Yao,^3^ Guo-Rong Xin,^4^ Xu-Wen Li^1^ & Yue-Wei Guo[[1]](#footnote-2)

[**S1.** The ^1^H NMR spectrum of **1** (CDCl_3_, 400 MHz) 4](#__RefHeading___Toc362858440)

[**S2.** The ^13^C NMR spectrum of **1** (CDCl3, 100 MHz) 5](#__RefHeading___Toc362858441)

[**S3.** The HSQC spectrum of **1** (CDCl3) 6](#__RefHeading___Toc362858442)

[**S4.** The COSY spectrum of **1** (CDCl_3_) 7](#__RefHeading___Toc362858443)

[**S5.** The HMBC spectrum of **1** (CDCl_3_) 8](#__RefHeading___Toc362858444)

[**S6.** The ROESY spectrum of **1** (CDCl_3_, 600MHz) 9](#__RefHeading___Toc362858445)

[**S7.** The HRESI spectrum of **1** 10](#__RefHeading___Toc362858446)

[**S8.** The ^1^H NMR spectrum of **2** (CDCl_3_, 400 MHz) 11](#__RefHeading___Toc362858447)

[**S9.** The ^13^C NMR spectrum of **2** (CDCl_3_, 100 MHz) 12](#__RefHeading___Toc362858448)

[**S10.** The HSQC spectrum of **2** (CDCl_3_) 13](#__RefHeading___Toc362858449)

[**S11.** The COSY spectrum of **2** (CDCl_3_) 14](#__RefHeading___Toc362858450)

[**S12.** The HMBC spectrum of **2** (CDCl_3_) 15](#__RefHeading___Toc362858451)

[**S13.** The ROSY spectrum of **2** (CDCl_3_, 600MHz) 16](#__RefHeading___Toc362858452)

[**S14.** The HRESI spectrum of **2** 17](#__RefHeading___Toc362858453)

[**S15.** The ^1^H NMR spectrum of **3** (CDCl_3_, 400 MHz) 18](#__RefHeading___Toc362858454)

[**S16.** The ^13^C NMR spectrum of **3** (CDCl_3_, 100 MHz) 19](#__RefHeading___Toc362858455)

[**S17.** The HRESI spectrum of **3** 20](#__RefHeading___Toc362858456)

[**S18.** The ^1^H NMR spectrum of **4** (CDCl_3_, 400 MHz) 21](#__RefHeading___Toc362858457)

[**S19.** The ^13^C NMR spectrum of **4** (CDCl_3_, 100 MHz) 22](#__RefHeading___Toc362858458)

[**S20.** The HSQC spectrum of **4** (CDCl_3_) 23](#__RefHeading___Toc362858459)

[**S21.** The COSY spectrum of **4** (CDCl_3_) 24](#__RefHeading___Toc362858460)

[**S22.** The HMBC spectrum of **4** (CDCl_3_) 25](#__RefHeading___Toc362858461)

[**S23.** The ROESY spectrum of **4** (CDCl_3_, 600MHz) 26](#__RefHeading___Toc362858462)

[**S24.** The HREI spectrum of **4** 27](#__RefHeading___Toc362858463)

[**S25.** The HREI spectrum of **4** 28](#__RefHeading___Toc362858464)

[**S26.** The ^1^H NMR spectrum of **5** (CDCl_3_, 400 MHz) 29](#__RefHeading___Toc362858465)

[**S27.** The ^13^C NMR spectrum of **5** (CDCl_3_, 100 MHz) 30](#__RefHeading___Toc362858466)

[**S28.** The HRESI spectrum of **5** 31](#__RefHeading___Toc362858467)

[**S29.** The ^1^H NMR spectrum of **6** (CDCl_3_, 400 MHz) 32](#__RefHeading___Toc362858468)

[**S30.** The 13C NMR spectrum of **6** (CDCl3, 100 MH 33](#__RefHeading___Toc362858469)

[**S31.** The HSQC spectrum of **6** (CDCl_3_) 34](#__RefHeading___Toc362858470)

[**S32.** The COSY spectrum of **6** (CDCl_3_) 35](#__RefHeading___Toc362858471)

[**S33.** The HMBC spectrum of **6** (CDCl_3_) 36](#__RefHeading___Toc362858472)

[**S34.** The ROESY spectrum of **6** (CDCl_3_, 600MHz) 37](#__RefHeading___Toc362858473)

[**S35.** The HRESI spectrum of **6** 38](#__RefHeading___Toc362858474)

[**S36.** The ^1^H NMR spectrum of **7** (CDCl_3_, 400 MHz) 39](#__RefHeading___Toc362858475)

[**S37.** The ^13^C NMR spectrum of **7** (CDCl_3_, 100 MHz) 40](#__RefHeading___Toc362858476)

[**S38.** The HRESI spectrum of **7** 41](#__RefHeading___Toc362858477)

[**S39.** The ^1^H NMR spectrum of **8** (CDCl_3_, 400 MHz) 42](#__RefHeading___Toc362858478)

[**S40.** The ^13^C NMR spectrum of **8** (CDCl_3_, 100 MHz) 43](#__RefHeading___Toc362858479)

[**S41.** The HRESI spectrum of **8** 44](#__RefHeading___Toc362858480)

[**S42.** The ^1^H NMR spectrum of **9** (CDCl_3_, 400 MHz) 45](#__RefHeading___Toc362858481)

[**S43.** The ^13^C NMR spectrum of **9** (CDCl_3_, 100 MHz) 46](#__RefHeading___Toc362858482)

[**S44.** The HSQC spectrum of **9** (CDCl_3_) 47](#__RefHeading___Toc362858483)

[**S45.** The COSY spectrum of **9** (CDCl_3_) 48](#__RefHeading___Toc362858484)

[**S46.** The HMBC spectrum of **9** (CDCl_3_) 49](#__RefHeading___Toc362858485)

[**S47.** The ROESY spectrum of **9** (CDCl_3_, 600 MHz) 50](#__RefHeading___Toc362858486)

[**S48.** The HRESI spectrum of **9** 51](#__RefHeading___Toc362858487)

# **S1.** The ^1^H NMR spectrum of **1** (CDCl_3_, 400 MHz)

# **S2.** The ^13^C NMR spectrum of **1** (CDCl3, 100 MHz)

# **S3.** The HSQC spectrum of **1** (CDCl3)

# **S4.** The COSY spectrum of **1** (CDCl_3_)

# **S5.** The HMBC spectrum of **1** (CDCl_3_)

# **S6.** The ROESY spectrum of **1** (CDCl_3_, 600MHz)

# **S7.** The HRESI spectrum of **1**

# **S8.** The ^1^H NMR spectrum of **2** (CDCl_3_, 400 MHz)

# **S9.** The ^13^C NMR spectrum of **2** (CDCl_3_, 100 MHz)

# **S10.** The HSQC spectrum of **2** (CDCl_3_)

# **S11.** The COSY spectrum of **2** (CDCl_3_)

# **S12.** The HMBC spectrum of **2** (CDCl_3_)

# **S13.** The ROSY spectrum of **2** (CDCl_3_, 600MHz)

# **S14.** The HRESI spectrum of **2**

# **S15.** The ^1^H NMR spectrum of **3** (CDCl_3_, 400 MHz)

# **S16.** The ^13^C NMR spectrum of **3** (CDCl_3_, 100 MHz)

# **S17.** The HRESI spectrum of **3**

# **S18.** The ^1^H NMR spectrum of **4** (CDCl_3_, 400 MHz)

# **S19.** The ^13^C NMR spectrum of **4** (CDCl_3_, 100 MHz)

# **S20.** The HSQC spectrum of **4** (CDCl_3_)

# **S21.** The COSY spectrum of **4** (CDCl_3_)

# **S22.** The HMBC spectrum of **4** (CDCl_3_)

# **S23.** The ROESY spectrum of **4** (CDCl_3_, 600MHz)

# **S24.** The HREI spectrum of **4**

# **S25.** The HREI spectrum of **4**

# **S26.** The ^1^H NMR spectrum of **5** (CDCl_3_, 400 MHz)

# **S27.** The ^13^C NMR spectrum of **5** (CDCl_3_, 100 MHz)

# **S28.** The HRESI spectrum of **5**

# **S29.** The ^1^H NMR spectrum of **6** (CDCl_3_, 400 MHz)

**S30.** The 13C NMR spectrum of **6** (CDCl3, 100 MHz)

# **S31.** The HSQC spectrum of **6** (CDCl_3_)

# **S32.** The COSY spectrum of **6** (CDCl_3_)

# **S33.** The HMBC spectrum of **6** (CDCl_3_)

# **S34.** The ROESY spectrum of **6** (CDCl_3_, 600MHz)

# **S35.** The HRESI spectrum of **6**

# **S36.** The ^1^H NMR spectrum of **7** (CDCl_3_, 400 MHz)

# **S37.** The ^13^C NMR spectrum of **7** (CDCl_3_, 100 MHz)

# **S38.** The HRESI spectrum of **7**

# **S39.** The ^1^H NMR spectrum of **8** (CDCl_3_, 400 MHz)

# **S40.** The ^13^C NMR spectrum of **8** (CDCl_3_, 100 MHz)

# **S41.** The HSQC spectrum of **8** (CDCl_3_)

# **S42.** The COSY spectrum of **8** (CDCl_3_)

# **S43.** The HMBC spectrum of **8** (CDCl_3_)

# **S44.** The ROESY spectrum of **8** (CDCl_3_, 600 MHz)

# **S45.** The HRESI spectrum of **8**

1. ^1^State Key Laboratory of Drug Research, Shanghai Institute of Materia Medica, Chinese Academy of Sciences, Shanghai, 201203, China

   ^2^Department of Organic Chemistry, University of Debrecen, POB 20, 4010 Debrecen, Hungary

   ^3^Syngenta Jealott’s Hill International Research Centre, Berkshire RG42 6EY, United Kingdom

   ^4^Institute of Biological Science, Sun Yat-Sen University, Xin Gang West Road 135, Guangzhou 510275, China

   Correspondence and requests for materials should be addressed to X.-W. L. (email: xwli@simm.ac.cn) or Y.-W. G. (email: ywguo@simm.ac.cn). [↑](#footnote-ref-2)
